# High expression of *SMARCA4* or *SMARCA2* is frequently associated with an opposite prognosis in cancer

**DOI:** 10.1038/s41598-018-20217-3

**Published:** 2018-02-01

**Authors:** Jose A. Guerrero-Martínez, Jose C. Reyes

**Affiliations:** Centro Andaluz de Biología Molecular y Medicina Regenerativa-CABIMER, Consejo Superior de Investigaciones Científicas-Universidad de Sevilla-Universidad Pablo de Olavide (CSIC-USE-UPO). Av. Americo Vespucio 24, 41092 Seville, Spain

## Abstract

The gene encoding the ATPase of the chromatin remodeling SWI/SNF complexes *SMARCA4* (*BRG1*) is often mutated or silenced in tumors, suggesting a role as tumor suppressor. Nonetheless, recent reports show requirement of SMARCA4 for tumor cells growth. Here, we performed a computational meta-analysis using gene expression, prognosis, and clinicopathological data to clarify the role of SMARCA4 and the alternative SWI/SNF ATPase SMARCA2 (BRM) in cancer. We show that while the *SMARCA4* gene is mostly overexpressed in tumors, *SMARCA2* is almost invariably downexpressed in tumors. High *SMARCA4* expression was associated with poor prognosis in many types of tumors, including liver hepatocellular carcinoma (LIHC), and kidney renal clear cell carcinoma (KIRC). In contrast, high *SMARCA2* expression was associated with good prognosis. We compared tumors with high versus low expression of *SMARCA4* or *SMARCA2* in LIHC and KIRC cohorts from The Cancer Genome Atlas. While a high expression of *SMARCA4* is associated with aggressive tumors, a high expression of *SMARCA2* is associated with benign differentiated tumors, suggesting that SMARCA4 and SMARCA2 play opposite roles in cancer. Our results demonstrate that expression of *SMARCA4* and *SMARCA2* have a high prognostic value and challenge the broadly accepted general role of SMARCA4 as a tumor suppressor.

## Introduction

ATP-dependent chromatin remodeling is essential for almost every aspect of DNA metabolism including transcription, recombination, DNA repair, and DNA replication^1,2^. Therefore, it is not surprising that chromatin remodeling enzymes play a fundamental role in the development of cancer^3,4^. The first chromatin remodeling machinery identified was the SWI/SNF complex (also called the BAF complex), comprised in mammals by 11–15 subunits^5–7^. In fact, there is not a single SWI/SNF complex but rather a polymorphic family of complexes that also includes different members of small gene families. The enzymatic motor of the complexes are two mutually exclusive ATPases of the SNF2 family called SMARCA2 (also called BRAHMA, BRM)^8^ and SMARCA4 (also called BRAHMA RELATED GENE 1, BRG1)^9^. The mammalian SWI/SNF complexes have been involved in chromatin remodeling at enhancers, promoters, and gene bodies and are associated with gene activation and repression (see for example^10–15^). In addition, members of the SWI/SNF complexes have been implicated in DNA repair, and genome instability^16^. Importantly, genes encoding subunits of the SWI/SNF complexes are mutated in about 20% of all human tumor samples, making them among the most frequently mutated complexes in cancer^7,17–20^. The mechanisms by which loss-of-function mutations in SWI/SNF complex subunits trigger tumor formation or affect tumor cell behavior is still a highly debated issue. Several data point to the pathological effects of aberrant residual SWI/SNF complexes as the cause of the potential selective advantage of SWI/SNF mutant cancer cells^21–24^.

The human *SMARCA4* gene is frequently mutated in ovarian small cell carcinoma of the hypercalcemic type (in approx. 90% of the cases)^25–27^, and at much lower frequency in other cancer types^28–32^. In addition, *SMARCA4* has been found to be silenced or mutated in a number of cancer cell lines^33–35^. *Brg1* homozygous knockout mice die early during development; however, heterozygote mice or conditional inactivation of *Brg1* in some adult tissues display increased tumor formation^36–38^. While *SMARCA2* is not frequently mutated in tumors, it has been found to be silenced in a number of cancer cell lines^39^ and primary tumors^33,40^. *Brm* knockout mice develop normally, but *Brm*^−/−^ embryonic fibroblasts present increased proliferation *in vitro*^41^. Furthermore, heterozygote and homozygote *Brm* mutants treated with carcinogens display increased tumor development^39^. Re-expression of SMARCA4 or SMARCA2 into cancer cell lines deficient for these proteins decreases cell proliferation^34,42,43^. Taken together, these results indicate that SMARCA4 and SMARCA2 have tumor suppressor activity. However, other recent reports point to essential roles of SMARCA4 and/or SMARCA2 in cell survival and proliferation in some types of cancers^12,44–46^, complicating our understanding of the role of these ATPases in cancer. In order to clarify this complex scenario, we have now used data from The Cancer Genome Atlas (TCGA) and other databases to investigate the levels of *SMARCA4* and *SMARCA2* mRNAs in several types of cancer. Notably, while *SMARCA4* was mostly overexpressed in tumors, *SMARCA2* expression decreased in tumors, as compared to normal tissue. A meta-analysis of prognosis data indicated that tumors with high *SMARCA4* expression are mostly associated with poor prognosis, while tumors with high *SMARCA2* expression are mostly associated with good prognosis. Analyzing liver hepatocellular carcinoma and kidney renal clear cell carcinoma TCGA cohorts, we found that high levels of *SMARCA4* and *SMARCA2* transcripts were inversely associated with survival prognosis, clinicopathological factors, and gene expression patterns, pointing to an inverse role of *SMARCA4* and *SMARCA2* in cancer.

## Results

### *SMARCA4* is overexpressed, and *SMARCA2* is underexpressed, in multiple types of tumors

**Figure 1:**
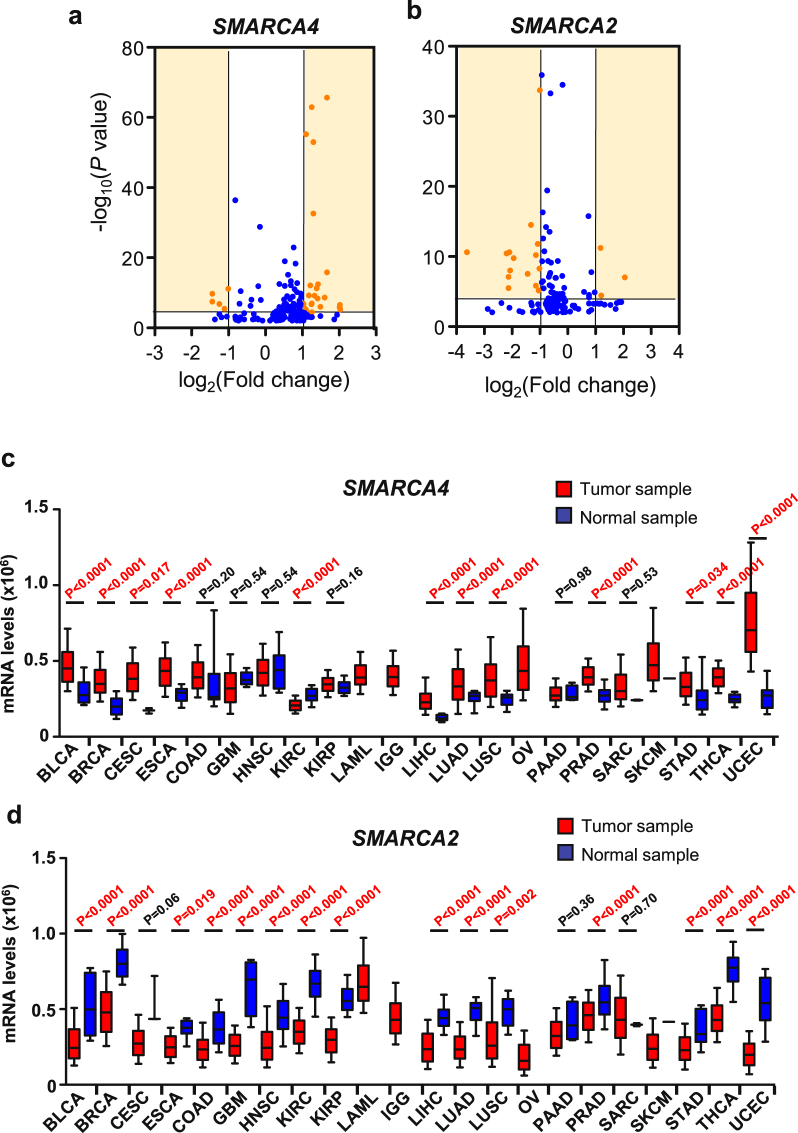
*SMARCA4* is overexpressed, and *SMARCA2* is underexpressed, in multiple types of tumors. (**a**,**b**) Volcano plots of *SMARCA4* (**a**) or *SMARCA2* (**b**) expression change (log_2_(FC)) in tumors with respect to normal samples versus significance (−log_10_(*P* value)), from different datasets. Data were obtained from ONCOMINE. Datasets with changes of *P* > 0.01 were not included. Highly significant changes (*P* ≤ 0.0001 and |FC| ≥ 2) are highlighted in orange and listed in Supplementary Tables S1 and S2. (**c**,**d**) Boxplot of levels of *SMARCA4* (**c**) or *SMARCA2* (**d**) mRNA (RNA-seq data) in normal or tumor samples of 22 different tumor cohorts from TCGA. Names and number of tumors and normal samples are listed in Supplementary Table S3. Significant Student’s t-test *P* value (*P* ≤ 0.05) are depicted in red.

First we compared the levels of *SMARCA4* and *SMARCA2* transcripts in normal tissue with respect to tumor tissue in different types of tumors. For this, we performed a meta-analysis of microarray expression data from different studies collected form the ONCOMINE database^47^. In 130 out of the 161 datasets selected (see methods), *SMARCA4* was found to be more highly expressed in the tumor samples than in the normal samples (Fig. 1a). Of the 32 datasets with highly significant changes (*P* ≤ 0.0001 and |lineal fold change (FC)| ≥ 2) (Fig. 1a; Supplementary Table S1), 26 (81%) presented higher levels of *SMARCA4* transcript in tumor samples than in non-tumor samples. A similar analysis for *SMARCA2* showed that its expression was reduced in 104 out of the 132 tumor datasets selected. Of the 16 datasets with highly significant changes (*P* ≤ 0.0001 and |FC| ≥ 2) (Fig. 1b and Supplementary Table S2), 13 (81%) presented lower levels of *SMARCA2* transcript in tumor samples than in non-tumor samples. These data suggest that *SMARCA4* gene is mostly overexpressed in tumors while, in contrast, *SMARCA2* is mostly underexpressed in tumors. In order to corroborate these results, we collected total RNA-seq normalized data of 22 different types of tumors from The Cancer Genome Atlas (TCGA) consortium (Supplementary Table S3). Levels of *SMARCA4* transcript were significantly higher in tumors than in normal tissue for 11 tumor types, and either unchanged or not quantifiable (due to lack of normal samples) in a further 10 tumor types (Fig. 1c). Only one type of tumor—kidney renal clear cell carcinomas (KIRC) — showed a higher expression of *SMARCA4* in normal samples than in tumor samples. In stark contrast, *SMARCA2* transcript levels were decreased in tumors compared to normal samples in 15 of the 22 tumor types analyzed and was not overexpressed in any type of tumor (Fig. 1d). These data confirm that *SMARCA4* is mostly overexpressed in tumors, while *SMARCA2* is mostly underexpressed in tumors.

We next investigated whether *SMARCA4* overexpression occurs predominantly in tumors harboring *SMARCA4* mutations as a possible consequence of a putative negative autoregulation. Using data on *SMARCA4* mutations in 18 types of tumors obtained from TCGA through cBioPortal^48^, we found *SMARCA4* to be mutated in either none or up to 8.5% of the samples, depending on the tumor type. *SMARCA4* mutated tumors displayed similar level of accumulation of *SMARCA4* mRNA as tumors harboring non-mutated *SMARCA4* (Supplementary Fig. S1a). Similar results were found for *SMARCA2* (Supplementary Fig. S1b). Taken together, these data demonstrate that *SMARCA4* expression is upregulated and *SMARCA2* is downregulated in most types of tumors irrespectively of the presence of mutations in the gene.

### Inverse association for prognosis between high expression of *SMARCA4* and *SMARCA2*

**Figure 2:**
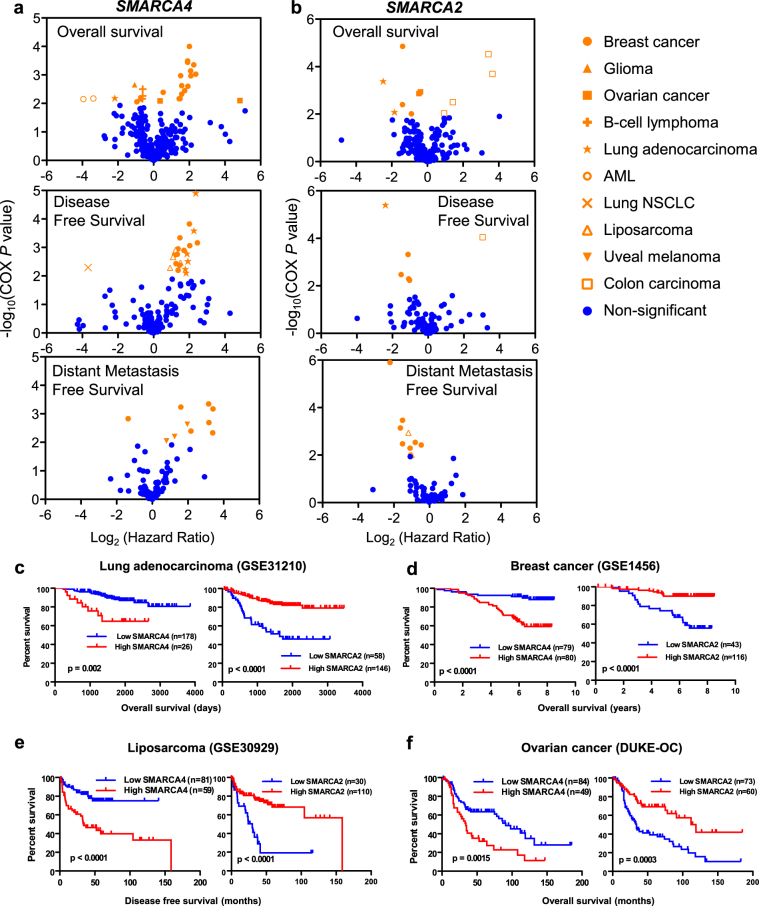
Meta-analysis of prognosis consequences of *SMARCA4* or *SMARCA2* upregulation. (**a**,**b**) Cox regression analysis of the correlation between SMARCA4 (**a**) or SMARCA2 (**b**) tumor expression levels and patient survival in different studies (data collected from PrognoScan). Volcano plots of log_2_(HR) versus significance (−log_10_(COX *P* value)) from different datasets are shown. A positive log_2_(HR) value indicates that the analyzed risk increases when the level of gene expression increases, and thus the prognosis is worse. Conversely, a negative Log_2_(HR) value implies a better prognosis for patients with tumors with higher values of gene expression. Different survival endpoints (overall survival, disease-free survival, and distant metastasis–free survival) are shown in different graphics. Significant changes (COX *P* ≤ 0.01) are highlighted in orange. (**c–f**) Kaplan-Meier plots showing inverse prognosis behavior of *SMARCA4* and *SMARCA2* expression in the same studies. Data corresponding to four different types of tumors are shown: lung adenocarcinoma^104^, breast cancer^105^, liposarcoma^106^, and ovarian cancer^107^. GEO references of the data are provided when available. Long-rank test *P* values are provided. Patient tumors were divided into two expression groups (high and low) according to PrognoScan (see Methods).

We next investigated whether having tumors with increased expression of *SMARCA4* or *SMARCA2* was linked to patient prognosis. For this, we performed a meta-analysis of data collected from the PrognoScan database^49^. This database allows systematic analysis of the prognostic value of the expression of a gene across a large collection of publicly available cancer microarray datasets. Correlation between gene expression and patient prognosis was evaluated using COX univariate analysis. Volcano plots of log_2_ hazard ratios (HR) versus −log_10_(COX *P* value) were drawn for every endpoint available (overall survival, disease-free survival, and distant metastasis-free survival). High expression of *SMARCA4* was significantly associated (COX *P* ≤ 0.01) with a poor prognosis in breast and ovarian cancer, lung adenocarcinoma, liposarcoma and uveal melanoma datasets (Fig. 2a). In contrast, high expression of *SMARCA2* was associated to good prognosis in breast and ovarian cancer, lung adenocarcinoma, and liposarcoma datasets (Fig. 2b). In fact, high expression of SMARCA2 was associated with poor prognosis only in colon carcinoma. Kaplan-Meier survival plots of patients from the same dataset, with high versus low expression of *SMARCA4* and *SMARCA2* are shown in Fig. 2c–f. These data indicate that, at least in some types of tumors, upregulation of *SMARCA4* or *SMARCA2* has opposite consequences in prognosis.

**Figure 3:**
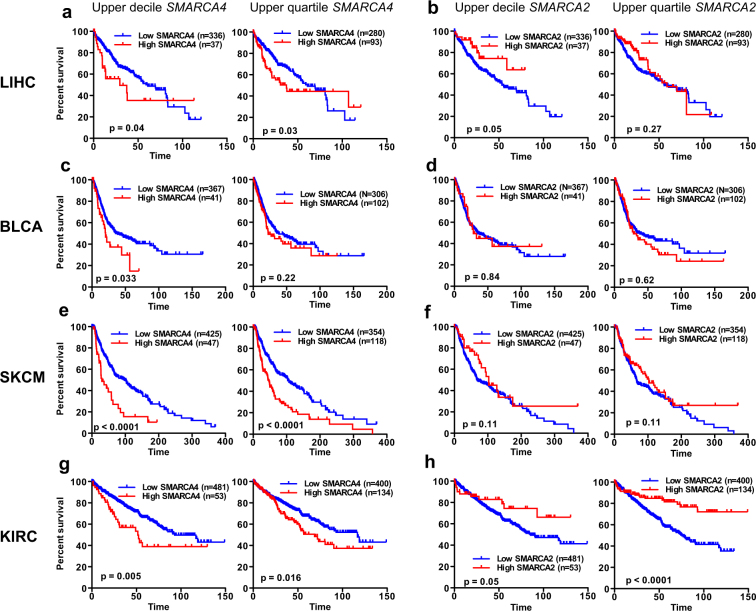
High levels of *SMARCA4* or *SMARCA2* expression are frequently associated with the opposite prognosis. Kaplan Meier survival plots of patients with tumors that have high or low expression levels of *SMARCA4* (**a**,**c**,**e**,**g**) or *SMARCA2* (**b**,**d**,**f**,**h**) in liver hepatocellular carcinoma (LIHC), bladder urothelial carcinoma (BLCA), skin cutaneous melanoma (SKCM), or kidney renal clear cell carcinoma (KIRC) cohorts from TCGA. Patients with tumors that had expression values in the upper decile (left panels) or upper quartile (right panels) values were compared with the rest of the patients.

Next, we extended these studies to other types of tumors using TCGA data. Clinical data of cohorts of four different types of tumors were collected: liver hepatocellular carcinoma (LIHC)^50^, bladder urothelial carcinoma (BLCA)^51^, skin cutaneous melanoma (SKCM)^52^, kidney renal clear cell carcinoma (KIRC)^53^. Tumor collections were ranked according to the *SMARCA4* or *SMARCA2* mRNA levels (RNA-seq data). Then, survival of patients with expression values in the upper decile (first and third columns, Fig. 3) or upper quartile (second and fourth columns, Fig. 3) were compared with survival of the remaining patients. Analysis of these plots indicates that high expression of *SMARCA4* is associated with poor prognosis in LIHC, BLCA, SKCM and KIRC (Fig. 3a,c,e,g). In clear contrast, high expression of *SMARCA2* is associated with good prognosis in LIHC and KIRC (Fig. 3b,h) while in SKCM, prognosis improved but not significantly (Fig. 3f). Taken together, these data suggest that in most of the cohorts analyzed, high expression of *SMARCA4* is associated with poor prognosis, while high expression of *SMARCA2* is associated with good prognosis.

**Figure 4:**
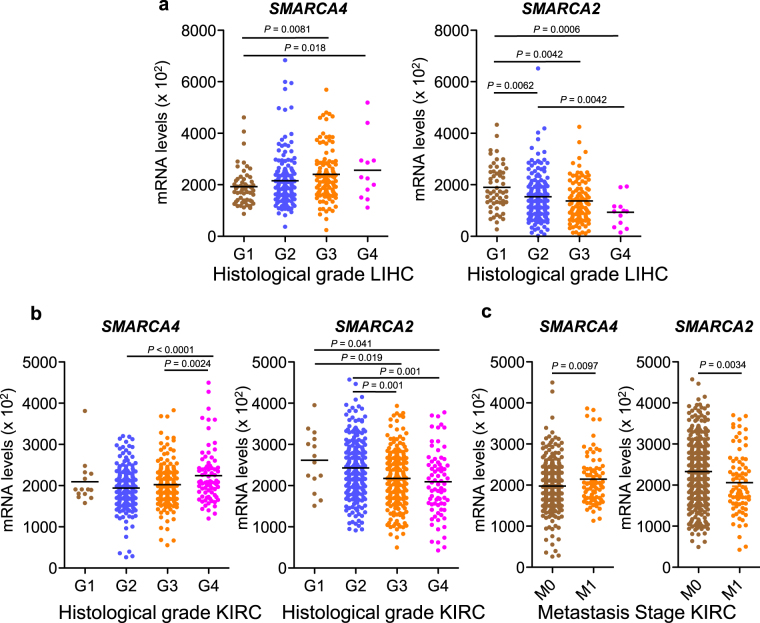
Correlation between *SMARCA4* or *SMARCA2* expression levels and clinicopathological factors. (**a**) Boxplot of levels of *SMARCA4* (left panel) and *SMARCA2* (right panel) transcript (normalized RNA-seq data) in LIHC tumors with different histological grades (G1 to G4). (**b**) Boxplot of transcript levels of *SMARCA4* (left panel) or *SMARCA2* (right panel) in KIRC tumors with different histological grades (G1 to G4). (**c**) Boxplot of transcript levels of *SMARCA4* (left panel) or *SMARCA2* (right panel) in KIRC tumors with different metastasis stages (M0 or M1). See Methods for description of G and M grading.

We further investigated the apparently opposite roles of *SMARCA4* and *SMARCA2* in the LIHC and KIRC tumors types. First we investigated whether expression levels of the *SMARCA4* and *SMARCA2* genes were associated to specific clinicopathological factors such as gender, age, tumor stage (T1 to T4 and stage I to IV), lymph node metastasis (N), distant metastasis (M), and differentiation (histological grade G1 to G4), obtained from TCGA. Consistently with the prognosis results, LIHC tumors with high levels of *SMARCA4* expression (upper decile) presented a significant increased proportion of advanced stages, and poorly differentiated histology with respect to the rest of the LIHC tumors analyzed (Table 1). In contrast, tumors with high levels of *SMARCA2* transcript (upper decile) presented increased proportion of well-differentiated tumors (Table 1). Further, increased undifferentiated histological grade is associated with a progressive increase of *SMARCA4* and a decrease of *SMARCA2* expression (Fig. 4a). Similarly, in KIRC tumors, high expression of *SMARCA4* is associated with increased undifferentiated histological grade (Fig. 4b and Table 2), while high levels of *SMARCA2* were associated with low tumor stages and well differentiated histology (Fig. 4b and Table 2). In addition, in KIRC tumors, high expression of *SMARCA4* was strongly associated with the presence of metastasis (high proportion of N1, *P* = 0.035, and M1, *P* = 0.0009) (Table 2). In stark contrast, this trend was not observed in tumors with a high expression of *SMARCA2*. In fact, metastatic KIRC tumors (M1) presented significantly increased levels of *SMARCA4* mRNA and decreased levels of *SMARCA2* mRNA, with respect to non-metastatic tumors (Fig. 4c).

**Table 1:** Association between *SMARCA4* and *SMARCA2* mRNA expression and clinicopathological factors in the liver hepatocellular carcinoma dataset from TCGA.

Factor	*SMARCA4* expression			*SMARCA2* expression		
	High expression^a^ n = 37	Lower expression^b^ n = 335	*P* value	High expression^a^ n = 37	Lower expression^b^ n = 335	*P* value
Age	57.4 ± 13.5	59.7 ± 13.5	0.30^c^	60.8 ± 12.2	59.3 ± 13.6	0.49^c^
Gender			**0.01** ^d^			0.70^d^
Male	18 (48.6%)	233 (69.6%)		24 (64.9%)	227 (67.8%)	
Female	19 (51.4%)	102 (30.4%)		13 (35.1%)	108 (32.2%)	
Tumor Stage (T)			**0.008** ^d^			0.29^d^
T1 + T2	21 (56.8%)	255 (76.8%)		25 (67.6%)	251 (75.6%)	
T3 + T4	16 (43.2%)	77 (23.2%)		12 (32.4%)	81 (24.4%)	
Tumor Stage (S)			**0.010** ^d^			0.11^d^
SI + SII	19 (55.9%)	239 (79.1%)		22 (62.9%)	236 (75.4%)	
SIII + SIV	15 (44.1%)	75 (23.9%)		13 (37.1%)	77 (24.6%)	
Lymph node metastasis			**0.014** ^d^			0.99^d^
N0	27 (93.1%)	226 (99.1%)		25 (96.2%)	228 (98.7%)	
N1	2 (6.9%)	2 (0.9%)		1 (3.8%)	3 (1.3%)	
Metastasis Stage Code			0.51^d^			0.27^d^
M0	30 (100%)	235 (98.3%)		24 (96.0%)	243 (98.8%)	
M1	0 (0.0%)	4 (1.7%)		1 (4.0%)	3 (1.2%)	
Histological grade			**0.004** ^d^			**0.019** ^d^
G1 + G2	15 (41.7%)	218 (65.9%)		30 (81.1%)	203 (61.5%)	
G3 + G4	21 (58.3%)	113 (34.1%)		7 (18.9%)	127 (38.5%)	

^a^Decile of the tumor population with higher levels of *SMARCA4* or *SMARCA2* mRNA.

^b^Rest of the tumors not included in the high expression decile.

^c^Student’s t-test.

^d^Chi-square test. Significant *P* values (*P* ≤ 0.05) are depicted in bold.

**Table 2:** Association between *SMARCA4* and *SMARCA2* mRNA expression and clinicopathological factors in the renal clear cell carcinoma dataset from TCGA.

Factor	*SMARCA4* expression			*SMARCA2* expression		
	High expression^a^ n = 54	Lower expression^b^ n = 479	*P* value	High expression^a^ n = 54	Lower expression^b^ n = 479	*P* value
Age	58.48 ± 11.0	60.84 ± 12.2	0.15^c^	57.6	60.9	0.06^c^
Gender			**0.037** ^d^			0.99^d^
Male	28 (51.9%)	317 (66.2%)		35 (64.8%)	310 (64.7%)	
Female	26 (48.1%)	162 (33.8%)		19 (35.2%)	169 (35.3%)	
Tumor stage			0.16^d^			**0.028** ^d^
T1 + T2	30 (55.6%)	312 (65.1%)		42 (75.9%)	300 (62.6%)	
T3 + T4	24 (44.4%)	167 (34.9%)		12 (24.1%)	179 (37.4%)	
Tumor stage (S)			0.076^d^			**0.019** ^d^
SI + SII	27 (50.0%)	297 (62.4%)		41 (75.9%)	283 (59.5%)	
SIII + SIV	27 (50.0%)	179 (37.6%)		13 (24.1%)	193 (40.5%)	
Lymph node metastasis			**0.035** ^d^			0.06^d^
N0	30 (85.7%)	209 (95.0%)		26 (92.9%)	213 (93.8%)	
N1	5 (14.3%)	11 (5.0%)		2 (7.1%)	14 (6.2%)	
Metastasis Stage Code			**0.0009** ^d^			0.24^d^
M0	34 (68.0%)	387 (86.0%)		45 (86.5%)	376 (83.9%)	
M1	16 (32.0%)	63 (14.0%)		7 (13.5%)	72 (16.1%)	
Histologic grade			**0.043** ^d^			**0.008** ^d^
G1 + G2	17 (32.7%)	224 (47.5%)		33 (63.5%)	209 (44.2%)	
G3 + G4	35 (66.0%)	248 (52.5%)		19 (36.5%)	264 (55.8%)	

^a^Decile of the tumor population with higher levels of *SMARCA4* or *SMARCA2* mRNA.

^b^Rest of the tumors not included in the high expression decile.

^c^Student’s t-test.

^d^Chi-square test. Significant *P* values (*P* ≤ 0.05) are depicted in bold.

### Transcriptome changes associated with *SMARCA4* or *SMARCA2* upregulation in liver hepatocellular carcinoma

**Figure 5:**
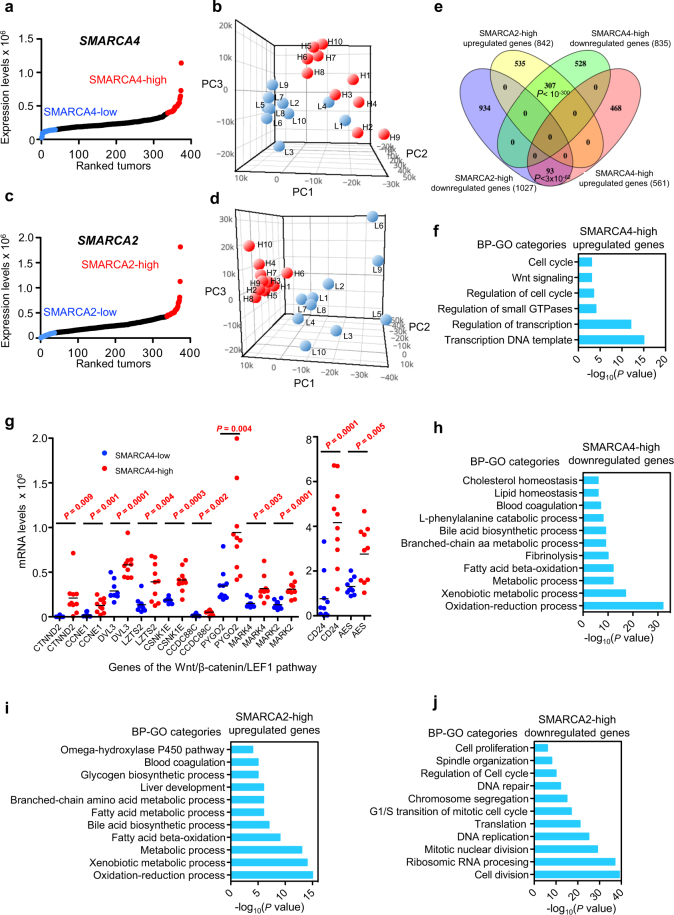
Transcriptome changes associated with *SMARCA4* or *SMARCA2* upregulation in LIHC. (**a**,**c**) Ranking (*x-axis*) of LIHC tumor samples according to *SMARCA4* (**a**) or *SMARCA2* (**c**) expression (normalized RNA-seq data) (*y-axis*). Dots corresponding to upper and lower deciles are depicted in red and blue, respectively. (**b**) Principal component (PC) analysis of transcriptomic data from ten SMARCA4-high (red) or ten SMARCA4-low (blue) tumors. (**d**) PC analysis of transcriptomic data from ten SMARCA2-high (red) or ten SMARCA2-low (blue) tumors. (**e**) Venn diagram showing overlap between SMARCA2-high upregulated and SMARCA4-high downregulated genes, and between SMARCA2-high downregulated and SMARCA4-high upregulated genes. Significance of overlap based on the hypergeometric test is provided. (**f**) Biological process (BP)-GO categories enriched in SMARCA4-high upregulated genes. (**g**) Expression levels (normalized RNA-seq data) of genes in the Wnt/β-catenin/LEF1 signal transduction pathway in SMARCA4-high and SMARCA4-low LIHC tumors. Student’s t-test *P* values are shown. (**h**) BP-GO categories enriched in SMARCA4-high downregulated genes. (**i**,**j**) BP-GO categories enriched in SMARCA2-high upregulated (**i**) and SMARCA2-high downregulated (**j**) genes. (**f**,**h–j**) Significance of the enrichment are presented as the *P* values of the hypergeometric test (−log_10_ transformed).

We next investigated the gene expression patterns that characterize LIHC tumors with high expression of *SMARCA4* or *SMARCA2*. For that, LIHC tumors were ranked according to the level of *SMARCA4* mRNA, and ten tumors were randomly selected from the upper decile (SMARCA4-high) or from the lower decile fractions (SMARCA4-low) (Fig. 5a; Supplementary Table S4). RNA-seq transcriptomic data of 60,483 genes from the 20 selected tumors were collected from TCGA and subjected to unsupervised principal component (PC) analysis. PC analysis differentiated almost all SMARCA4-low from SMARCA4-high tumors (only two tumors, L1 and L4, had an intermediate pattern), suggesting that the level of *SMARCA4* expression characterizes different subtypes of LIHC tumors (Fig. 5b). We then selected genes that were differentially expressed (*P* ≤ 0.01 and |FC| ≥ 2) in the SMARCA4-high versus the SMARCA4-low collection of tumors. Of the 1396 differentially expressed genes, 561 were upregulated and 835 were downregulated in SMARCA4-high tumors (Supplementary Fig. S2; Supplementary Table S5). A similar analysis was performed for SMARCA2-high versus the SMARCA2-low tumors (Fig. 5c; Supplementary Table S4). In this case, PC analysis clearly differentiated all SMARCA2-high from the SMARCA2-low tumors, being all SMARCA2-high tumors very closely related according to the three PCs analyzed (Fig. 5d). Differential expression analysis showed that 842 genes were significantly (*P* ≤ 0.01 and |FC| ≥ 2) upregulated and 1027 genes were downregulated, in SMARCA2-high versus SMARCA2-low tumors (Supplementary Fig. S2; Supplementary Table S6). Interestingly, the genes that were downregulated or upregulated in SMARCA4-high tumors strongly overlapped with the genes that were upregulated or downregulated, respectively, in SMARCA2-high tumors (Fig. 5e), demonstrating that these types of tumors not only have inverse prognosis but also opposite gene expression patterns.

Genes upregulated in SMARCA4-high tumors were enriched in regulation of transcription, cell cycle, DNA replication, and Wnt signaling pathways and functional categories (Fig. 5f; Supplementary Figs S3a and S4a). Furthermore, DNA binding sites for the LEF1 (*P* = 8.9 × 10^−18^), MAZ (*P* = 1.7 × 10^−12^), SP1 (*P* = 1.8 × 10^−10^) and E2F (*P* = 2.7 × 10^−10^) transcription factors were strongly overrepresented in the promoter regions of upregulated genes. In agreement with the enrichment in Wnt signaling categories and LEF1 DNA binding sites, several genes related to this pathway were activated (Fig. 5g). Genes downregulated in SMARCA4-high tumors were enriched in lipid and amino acids metabolism, xenobiotic metabolism, blood coagulation and aerobic respiration categories and pathways (Fig. 5h; Supplementary Figs S3b and S4a). All of these processes are important liver functions carried out by differentiated hepatocytes^54^. Consistently, promoters of downregulated genes were enriched in binding sites for typical liver transcription factors such as HNF1 (*P* = 9.1 × 10^−11^), FOXO4 (*P* = 5.9×10^−8^), and HNF4 (*P* = 6.4×10^−7^). These data suggest that SMARCA4-high tumors present a strong decrease of hepatocytes-specific functions, which is in agreement with the high proportion of undifferentiated cells (G3 + G4 histological grade) and the poor prognosis observed in these tumors (Table 1; Fig. 3a).

In contrast to SMARCA4-high tumors, SMARCA2-high tumors had upregulated gene sets prominently involved in hepatocytes-specific functions such as fatty acid metabolism, amino acid metabolism, drugs and xenobiotic metabolism, blood coagulation, and respiration categories and pathways (Fig. 5i; Supplementary Figs S3c and S4b). For instance, genes encoding typical hepatic enzymes such as tyrosine aminotransferase (*TAT*) and alcohol dehydrogenases 1B and 1 C (*ADH1B*, *ADH1C*) were increased 43.2-, 42.3- and 14.9-fold, respectively. In addition, genes downregulated in SMARCA2-high tumors were strongly enriched in ribosome RNA processing, translation, cell cycle, DNA-replication, and mitosis-related functions and pathways (Fig. 5j; Supplementary Figs S3d and S4b), and often presented E2F (*P* = 1.05×10^−26^), MYC (*P* = 3.9×10^−16^) and ELK1 (*P* = 1.2 × 10^−11^) binding sites, suggesting a reduced proliferation of these tumor cells. In sum, these data suggest that SMARCA2-high tumors maintain a high differentiation stage with low levels of proliferation, which is consistent with the good prognosis for patients with tumors with high levels of *SMARCA2* expression.

**Figure 6:**
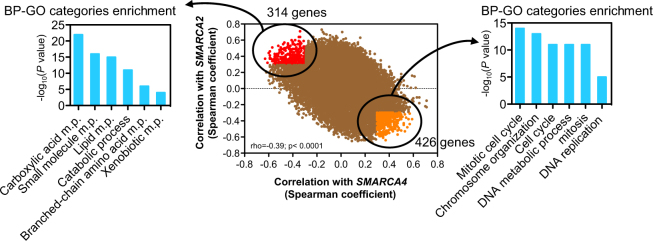
Analysis of genes co-expressed with SMARCA4 and SMARCA2 in LIHC. Scatter plot with each dot corresponding to a gene, with the x and y coordinates as the Spearman coexpression correlation coefficient of the gene with *SMARCA4* and *SMARCA2*, respectively (central panel). Spearman correlation coefficient (rho) and *P* values are shown. Genes that were more positively correlated with *SMARCA4* (rho ≥ 0.3) and negatively correlated with *SMARCA2* (rho ≤ −0.3) are depicted in orange. Genes that were more negatively correlated with *SMARCA4* (rho ≤ −0.3) and positively correlated with *SMARCA2* (rho ≥ 0.3) are depicted in red. Enrichment of GO categories is given for genes negatively correlated with *SMARCA4* and positively correlated with *SMARCA2* (left panels) and for genes positively correlated with *SMARCA4* and negatively correlated with *SMARCA2* (right panels). m.p. metabolic process.

In order to further characterize the roles of *SMARCA4* and *SMARCA2* in liver hepatocellular carcinoma, we have performed a genome-wide co-expression analysis using all available LIHC tumors from the TCGA cohort. We calculate Spearman correlation coefficients (rho) between the expression levels of *SMARCA4* or *SMARCA2* and 22,300 genes (data collected from cBioportal). A scatter plot was drawn with each dot corresponding to a gene, and the x and y coordinates as the Spearman correlation coefficient with *SMARCA4* and with *SMARCA2*, respectively. This showed a negative correlation (rho = −0.39; *P* < 0.0001) between Spearman coefficients, indicating that most of the genes whose expression is positively correlated with *SMARCA4* are negatively correlated with *SMARCA2*, and vice-versa (Fig. 6). We next analyzed the gene ontology (GO) of the genes that are most robustly co-regulated in an opposite way with *SMARCA4* and *SMARCA2* (rho ≥0.3 and ≤−0.3). Genes positively coexpressed with *SMARCA4* and negatively coexpressed with *SMARCA2* (Supplementary Table S7) were enriched in cell cycle (*CCNB1, CCNE1, CDK1, E2F1*), mitosis (*PLK, AURKB, CDC20, CDC25A*) and DNA replication (*POLD1, RFC4*) GO categories. In contrast, genes positively coexpressed with *SMARCA2* and negatively coexpressed with *SMARCA4* (Supplementary Table S7) were enriched in liver metabolism functions, such as lipid metabolism (*ACADL, ACSL1, LIPG*), amino acid metabolism (*TAT*, *BCKDHB, PAH, IDH1*), xenobiotic detoxification (*CYP3A4, CYP4V2, CYP8B1*), and blood coagulation (*F8, F11*) categories. This analysis confirms that high expression of *SMARCA4* or *SMARCA2* characterizes types of LIHC tumors with opposite patterns of gene expression.

### Transcriptome changes associated with *SMARCA4* or *SMARCA2* upregulation in kidney renal clear cell carcinoma

**Figure 7:**
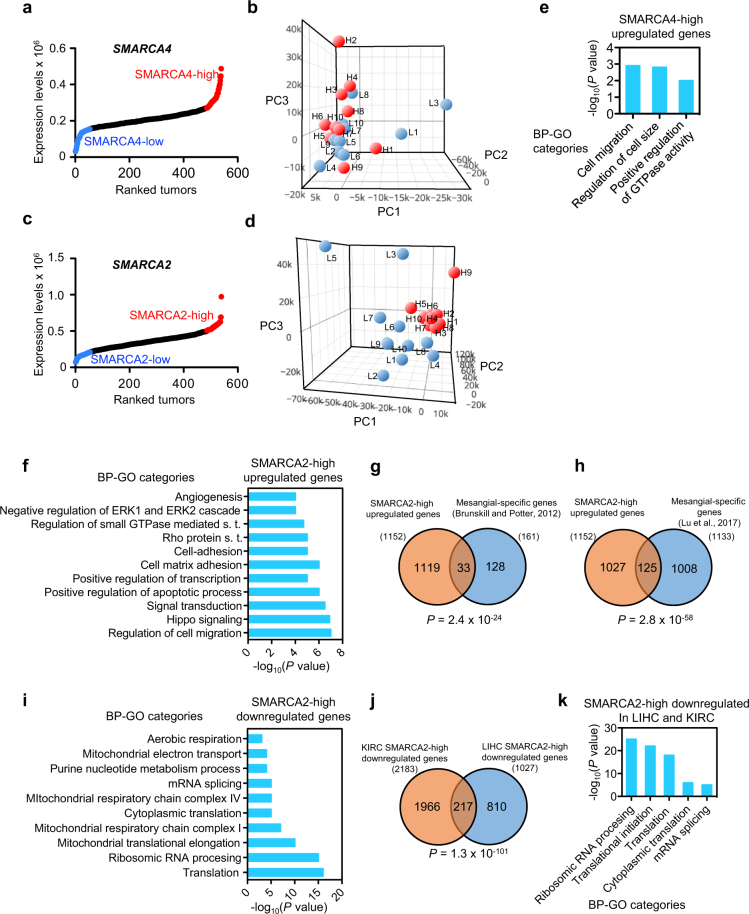
Transcriptome changes associated with *SMARCA4* or *SMARCA2* upregulation in KIRC. Ranking (*x-axis*) of KIRC tumor samples according to *SMARCA4* (**a**) or *SMARCA2* (**c**) expression (normalized RNA-seq data) (*y-axis*). Dots corresponding to the upper or lower decile are depicted in red or blue, respectively. (**b**) PC analysis of transcriptomic data from ten SMARCA4-high (red) and ten SMARCA4-low (blue) tumors. (**d**) PC analysis of transcriptomic data from ten SMARCA2-high (red) and ten SMARCA2-low (blue) tumors. (**e**,**f**) BP-GO categories enriched in SMARCA4-high (**e**) or SMARCA2-high (**f**) upregulated genes. s.t. signal transduction. (**g**,**h**) Overlapping between SMARCA2-high upregulated genes and mesangial cells-specific genes from two independent studies^66^: (**g**) and^67^ (GSE92650) (**h**). (**i**) BP-GO categories enriched in SMARCA2-high downregulated genes. (**j**) Overlap between SMARCA2-high downregulated genes from LIHC and KIRC tumors. (**g**,**h**,**j**) *P* values of the hypergeometric test. (**k**) BP-GO categories enriched in genes overlapping between SMARCA2-high upregulated genes from LIHC and from KIRC tumors. (**e**,**f**,**i**,**k**) Significance of the enrichments are given as the *P* values of the hypergeometric test (−log_10_ transformed).

A similar gene expression analysis as for LIHC tumors (above) was then performed for KIRC tumors. For this, ten tumors of each type: SMARCA4-high, SMARCA4-low, SMARCA2-high and SMARCA2-low, were selected from the KIRC cohort (Fig. 7a,c; Supplementary Table S4), and RNA-seq transcriptomic data from 60,483 genes were collected from TCGA. PC analysis of whole transcriptomic data was unable to discriminate between SMARCA4-high and SMARCA4-low tumors (Fig. 7b). However, PC analysis clearly differentiated SMARCA2-high from SMARCA2-low tumors (Fig. 7d), as SMARCA2-high tumors were very closely grouped with respect to the three PCs analyzed.

Only 140 genes were differentially expressed (*P* ≤ 0.01 and |FC| ≥ 2) in the SMARCA4-high versus the SMARCA4-low collection of tumors, of which 69 were upregulated and 71, downregulated (Supplementary Fig. S2; Supplementary Table S8). Upregulated genes were significantly enrichment in categories related to regulation of small GTP hydrolases (GTPases) (Fig. 7e; Supplementary Fig. S5a). Three genes encoding guanine nucleotide exchange factors (GEF) were upregulated: *ARHGEF18*, *RASGEF1A*, and *VAV2*. While ARHGEF18 and VAV2 are Rho GEFs, RASGEF1A is a regulator of RAP2. GEFs promote the transition of small GTPases from the inactive (GDP bound) to the active (GTP bound) state during signal transduction. Rho and RAP GTPases play essential roles in the regulation of cell morphology, cytokinesis, cell adhesion, and cell migration, and their activation and overexpression have been associated to metastasis^55,56^. Further, high expression of VAV2^57^, ARHGEF18^58^ and RASGEF1A^59^ has also been linked to metastasis, which is consistent with the high proportion of metastasis (M1 code) and metastatic lymph nodes (N1 code) we observed in SMARCA4-high tumors (Table 2). SMARCA4-high tumors also display high expression of *PAX8*, a well-known marker for primary and metastatic renal clear cell carcinomas^60,61^.

We observed 1152 genes to be upregulated, and 2183 genes downregulated, in the SMARCA2-high with respect to the SMARCA2-low tumors (Supplementary Fig. S2; Supplementary Table S9). SMARCA2-high upregulated genes were enriched in regulation of transcription, regulation of cell migration, regulation of small GTPases, focal adhesion, negative regulation of ERK1/2 cascade, and tight junction categories and pathways (Fig. 7f; Supplementary Figs S5b and S6a). Interestingly, several genes encoding Rho GTPase activating proteins (GAPs) were found to be upregulated in SMARCA2-high tumors, such as *DLC1* (FC = 3.8) *NF1* (FC = 2.3), *ARHGAP19* (FC = 2.1), *ARHGAP31* (FC = 3.0). This is in clear contrast with the presence of high levels of Rho GEFs in SMARCA4-high tumors. DLC1^62,63^ and NF1^64^ are well-known tumor suppressors, which is consistent with the good prognosis of the SMARCA2-high tumors. Increased expression levels of genes encoding cell-cell contact molecules (tight, GAP and adherens junctions) were also observed, including the *TJP1, JMY, JAM2, JAM3, GJA1*, and *OCLN* genes, suggesting a marked epithelial or endothelial phenotype of the SMARCA2-high tumors. Several genes encoding markers of endothelial cells were upregulated, such as *PCAM1, VWF, CD34, NRP1, TEK*, and *FLT1*, consistent with the fact that the renal glomerulus is mostly formed by three types of cells: endothelial cells, podocytes and mesangial cells^65^. Interestingly, we also observed a significant overlap between mesangial-expressed genes^66,67^ and genes upregulated in SMARCA4-high tumors (Fig. 7g,h). The fact that SMARCA2-high tumors express high levels of markers of glomerulus cell types is in agreement with the high differentiation (e.g., low histologic grade) of these tumors (Table 2).

Genes downregulated in SMARCA2-high tumors were strongly enriched in categories related to both cytosolic and mitochondrial ribosomal proteins, translation, and mitochondrial respiration electron transport (Fig. 7i; Supplementary Figs S5b and S6b). Promoters of downregulated genes were very significantly enriched in binding sites for ELK1 (*P* = 6.6 × 10^−45^), NRF1 (*P* = 1.4 × 10^−21^), NRF2 (*P* = 1.5 × 10^−9^) and MYC (*P* = 6.3 × 10^−15^). NRF1 and NRF2 have important functions controlling cell growth, respiration, mitochondrial DNA transcription and replication^68^. The downregulation of mitochondrial respiration suggested that SMARCA2-high tumors might present a strong Warburg effect^69^. However, none of the ten glycolysis genes (gene set M15109 from MSigDB) were upregulated in SMARCA2-high tumors, in fact the expression levels of *GAPDH* (FC = 0.47; *P* = 1.3×10^−5^) and *PFKFB4* (FC = 0.41; *P* = 0.0007) decreased, suggesting that SMARCA2-high tumors have a reduced energetic metabolism. Notably, analyzing for similarity between the transcription patterns of LIHC SMARCA2-high and KIRC SMARCA2-high tumors revealed a very significant overlap between downregulated genes in these two types of tumors (Fig. 7j). Most overlapping genes encoded ribosomal proteins and proteins related to translation and ribosome biogenesis (Fig. 7k). These data suggest that LIHC and KIRC tumors with high expression of *SMARCA2* have reduced translation and, therefore, probably a reduced cell growth.

## Discussion

### SMARCA4 is frequently upregulated in tumors

A role of SWI/SNF complexes as tumor suppressors is widely accepted, mostly based on the fact that genes encoding SWI/SNF subunits are mutated in a wide-ranging proportion of tumors^18,20^. Thus, *SMARCA4* is frequently mutated (more than 90% of the cases) in ovarian small cell carcinoma of the hypercalcemic type^25–27^. However, several studies and inspection of the TCGA data indicate that, in most of the tumor types *SMARCA4* mutations vary between 0% and 15% of the cases^18,20,25–32^. Tumor suppressor genes are normally either mutated or downregulated in tumor tissues^70^. However, we now show that *SMARCA4* is frequently overexpressed in tumors. Furthermore, we show that *SMARCA4* upregulation is associated with a poor prognosis in published datasets for breast and ovarian cancer, lung adenocarcinoma, liposarcoma, and uveal melanoma and in the SKCM, LIHC, BLCA, and KIRC TCGA cohorts, indicating that high expression of *SMARCA4* can be used as a prognosis marker for these types of tumors. Consistently, loss of expression of SMARCA4 protein has been recently associated to improved prognosis in clear cell renal cell carcinoma^71^. Increased expression of *SMARCA4* has been previously reported in several types of tumors^45,46,72–77^. In addition, several studies have shown that SMARCA4 is required for tumor cell proliferation^12,44–46^. Furthermore, we find that high levels of *SMARCA4* expression are associated with an advanced tumor stage and histological grade in LIHC, and with increased metastasis in KIRC. Taken together, these data suggest that, at least for several types of cancers, high expression of *SMARCA4* confers a selective advantage to tumor cells. This is, therefore, not consistent with a general role of SMARCA4 as a tumor suppressor. A context-dependent dual role of SMARCA4 in cancer has been also proposed by Dr. Imbalzano and collaborators^78^. A growing number of genes play both tumor suppressor or oncogenic roles in different tissue, tumor types or experimental settings^79^. Therefore, our data are not incompatible with a role of SMARCA4 as a tumor suppressor when it is mutated in certain types of tumors, probably due to the pathological activity of aberrant residual SWI/SNF complexes.

What is the mechanism by which increased levels of SMARCA4 are important for cancer development? The answer is still unclear but probably it is dependent on the cancer type. SMARCA4 has been shown to promote breast cancer by reprogramming lipid synthesis^45^ and to be required for maintaining repopulation of hematopoietic stem cells in leukemia^44^. In fact, a role of SMARCA4 in regulation of stem cells pluripotency has been well characterized^11,80^, and SMARCA4 is highly expressed in stem cells^81^. Therefore, it is possible that SMARCA4 plays an essential role in the maintenance of cancer stem cells. The role of the Wnt/β-catenin/LEF1 pathway in activation of hepatic cancer stem cells in hepatocellular carcinoma and during liver regeneration has been well characterized^82–84^. Interestingly, we observed that LIHC SMARCA4-high tumors presented increased levels of several genes of the Wnt/β-catenin/LEF1 pathway. This is also consistent with the relative undifferentiated state of these tumors, according to gene expression pattern and histological grade. In addition, we show that LIHC SMARCA4-high tumors had high expression of positive regulators of cell cycle progression and mitosis, such as cyclins, mitotic kinases, and DNA replication factors, which also suggests a positive correlation between levels of SMARCA4 and proliferation in LIHC. Consistently, Kaufmann *et al*., recently showed that knockdown of SMARCA4 impairs proliferation and decreases cyclin B and cyclin E expression in hepatocellular carcinoma cell lines^77^. A role of SWI/SNF complexes containing SMARCA4 in positive regulation of cell cycle genes^85^ has been previously described.

In KIRC SMARCA4-high tumors, we did not find increased expression of the Wnt/β-catenin/LEF1 pathway or cell cycle genes, illustrating the absence of similarity between SMARCA4-high tumors of different origins. However, we found that KIRC SMARCA4-high tumors presented a high proportion of metastasis. SMARCA4-high tumors displayed high expression of the RhoA GEFs *ARHGEF18* and *VAV2*, which are involved in activation of RhoA small GTPase. The RhoA signaling pathway and ARHGEF18 and VAV2 proteins have been implicated in metastasis formation^56–58^. Interestingly, RhoA signaling activation was reported upon SMARCA4 re-expression in SMARCA4-deficient human adrenal adenocarcinoma SW13 cells^86^.

### *SMARCA2* is frequently downregulated in tumors

In contrast to *SMARCA4*, *SMARCA2* expression was strongly downregulated in most cancer types, which is consistent with a role as tumor suppressor of this protein. *SMARCA2* levels were correlated with good prognosis in published datasets for breast and ovarian cancer, lung adenocarcinoma, and liposarcoma, and in the LIHC and KIRC TCGA cohorts. In addition, high levels of *SMARCA2* expression were associated with a low tumor stage and well-differentiated tumors in LIHC and KIRC. *SMARCA2* is not frequently mutated in tumors but gene silencing in tumor cell lines has been reported^33,39,43^. Several experimental data support a role of SMARCA2 as a tumor suppressor. *Brm*^−/−^ mouse embryonic fibroblasts present increased proliferation and have lost inhibition of growth by cell-cell contact^41^. Additionally, heterozygote and homozygote *Brm* mutant mice treated with carcinogens have increased tumor development^39,87^. Expression of *SMARCA2* is negatively regulated by mitogenic stimulation and Ras and ERK signaling, and restoration of SMARCA2 levels leads to reversion of the transformed phenotype^42,43,88^. Finally, SMARCA2 is not expressed in stem cells or during early development until the stage of blastocyst, and its levels increase during stem cells differentiation and during late development^41,81^. Recent data also show that SMARCA2 is required for cell cycle arrest during myoblast differentiation^89^. Taken together, these data suggest that a reduced level of *SMARCA2* expression confers a selective advantage for many types of tumor cells. In agreement with this, LIHC and KIRC SMARCA2-high tumors form a coherent and well-defined subtype of tumors, with high differentiation according to gene expression patterns and histological grade and with low expression of cell cycle genes (for LIHC) and low expression of ribosomal and translation genes (for both LIHC and KIRC). A reduction in the levels of *SMARCA2* transcript in a cohort of hepatocellular carcinomas has been previously reported^90^. In this study, SMARCA2 protein expression was lost in nine of 40 tumors and patients with these tumors presented a poor overall survival. Similarly, decrease of overall survival in SMARCA2 negative tumors has been also recently reported in clear cell renal cell carcinoma but only when levels of PBRM1 protein, another subunit of the SWI/SNF complex were also reduced^71,91^. These data suggest that *SMARCA2* expression is a good marker for characterizing LIHC and KIRC prognosis.

### Do SMARCA4 and SMARCA2 play antagonistic roles in cancer?

Our data demonstrate that levels of *SMARCA4* and *SMARCA2* expression correlate with opposite prognosis in several types of tumors and, in addition, with opposing clinicopathological factors and gene expression patterns in LIHC and KIRC tumors. Whether *SMARCA4* and *SMARCA2* expression are the cause or the consequence of differences in tumors is not yet clear. However, the facts that SMARCA4 expression is mostly associated to cell types that constantly undergo proliferation or self-renewal^81,92^ while SMARCA2 is absent from stem cells and inversely correlated with proliferation in several types of cells^42,88,89,92^, suggest the attractive possibility that the SWI/SNF complexes use a different ATPase, or a different ratio of ATPases, for proliferating-undifferentiated versus quiescent-differentiated conditions. How this equilibrium would be controlled is unclear. However, it has been demonstrated that a decrease in the protein level of one of the ATPases of the SWI/SNF complexes causes an increase of the level of the paralogous ATPase as well as its replacement in the complexes^41,93^, suggesting that the changes of the mRNA levels in tumors that we describe in this work can alter the composition of the complexes. Finally, it has been proposed that the SMARCA4 and SMARCA2 ATPases are appropriate targets for anticancer drugs design^94^. The antagonistic behavior uncovered in our work should be taken into account to design specific drugs that specifically target one but not the other ATPase.

## Methods

### Data collection and analysis of *SMARCA4* and *SMARCA2* levels

For meta-analysis of *SMARCA4* and *SMARCA2* transcript levels in normal and tumor samples data were collected from the cancer microarray expression database ONCOMINE 4.5^47^ (https://www.oncomine.org/*)*. Those datasets in which changes of expression between normal and tumor tissue were significant, with a *P* value ≤ 0.01 (Student’s t-test), and which ranked in the top 10% of the more significant changes, were selected. Volcano plots of −Log_10_(*P* value) versus log_2_(FC) were then generated.

Expression of *SMARCA4* and *SMARCA2* was also analyzed in 22 cohorts of different types of tumors from The Cancer Genome Atlas (TCGA) consortium. *SMARCA4* and *SMARCA2* RNA-seq mRNA expression data (FPKM-UQ normalized) in tumors and the corresponding available normal tissue samples were collected from TCGA (https://cancergenome.nih.gov/) though the Genomic Data Commons Data Portal (https://portal.gdc.cancer.gov). Types of tumors as well as the number of tumors and normal samples are provided in Supplementary Table S3.

All methods and use of data were carried out in accordance with relevant guidelines and regulations of the corresponding databases. No experiments were performed using human samples.

### Analysis of prognosis

Meta-analyses of the association of *SMARCA4* and *SMARCA2* expression levels with survival outcomes were performed using data collected from the PrognoScan database (http://www.abren.net/PrognoScan/)^49^. COX regression analysis^95^ data (HR and COX *P* value) were downloaded and used to construct volcano plots of log_2_(HR) versus −log_10_(COX *P* value) for every endpoint available (overall survival, disease-free survival and distant metastasis-free survival). Only studies with COX *P* values ≤ 0.01 were considered as significant. For Kaplan-Meier plots of Fig. 2c–f, patients were divided into two (high and low) groups according on the expression of *SMARCA4* or *SMARCA2* in the tumors. The optimal cut-point that gave the most pronounced corrected *P* value (in the log-rank test) between the two groups was provided by the PrognoScan database.

Association between *SMARCA4* or *SMARCA2* expression levels and prognosis was also analyzed in four cohorts of TCGA: Liver hepatocellular carcinoma (LIHC), bladder urothelial carcinoma (BLCA), skin cutaneous melanoma (SKCM), and kidney renal clear cell carcinoma (KIRC). Overall survival data of patients were collected from TCGA, and Kaplan-Meier plots were performed in Prism 5 (GraphPad). Significance was determined using log-rank test.

### Clinical data

Clinicopathological data of patients in the LIHC and KIRC cohorts were obtained from TGCA. For tumor description, the Tumor-Node-Metastasis (TNM) staging system (www.cancerstaging.org/) was used, whereby T followed by a number (1–4) describes the size of the tumor (with T4 being the largest); N followed by 1 or 0 indicates whether lymph nodes have metastasis or not, respectively; and M followed by 1 or 0 indicates whether the tumor has metastasized or not, respectively. Histopathologic grade G followed by a number (1–4) was also considered: G1, well differentiated; G2, moderately differentiated; G3, poorly differentiated and G4, undifferentiated tumor. We also considered the roman numeral stage annotation (S) from I to IV, with each number corresponding approximately to a combination of the TNM numbers. No subdivisions of stages were used (e.g., T1a, T1b, and T1c were considered as T1). To determine significance of differences between groups either Student’s t-test or Chi-square test with confidence interval of 95% were computed, using Prism 5 (GraphPad).

### Principal component and differential expression analysis

To characterize gene expression changes between tumors with high or low levels of SMARCA4 or SMARCA2, all analyzed LIHC or KIRC TCGA tumors were ranked according to their level of *SMARCA4* mRNA or *SMARCA2* mRNA, respectively. Ten tumors were then randomly selected from the upper decile (SMARCA4-high or SMARCA2-high) or from the lower decile (SMARCA4-low or SMARCA2-low) for each tumor type. These were used to generate following sets of ten tumors: SMARCA4-high LIHC, SMARCA4-low LIHC, SMARCA2-high LIHC, SMARCA2-low LIHC, SMARCA4-high KIRC, SMARCA4-low KIRC, SMARCA2-high KIRC, SMARCA2-low KIRC. The reference numbers of the TCGA tumors used is provided in Supplementary Table S4. The expression patterns of SMARCA4-high versus SMARCA4-low, and SMARCA2-high versus SMARCA2-low, in both types of tumors were then subjected to PC analysis. For this, RNA-seq expression data (FPKM-UQ normalized) of 60,483 Ensembl reference genes from the 20 compared samples were centered on the median (z-scores), and non-expressed genes in any of the samples were removed. Data were then subjected to unsupervised PC analysis using default parameters in MultiExperiment viewer (MeV) 4.8.1 software^96^. Data of the three PCs (PC1, PC2 and PC3) were then represented in 3D scatter plots using Plotly 2.0(https://plot.ly/create/).

Differential expression analyses between sets of tumors (SMARCA4-high LIHC versus SMARCA4-low LIHC; SMARCA2-high LIHC versus SMARCA2-low LIHC; SMARCA4-high KIRC versus SMARCA4-low KIRC; SMARCA2-high KIRC versus SMARCA2-low KIRC) were performed using RNA-seq expression data (FPKM-UQ normalized) of 60483 Ensemble genes and standard methods^97^. Unpaired two-samples Students t-test with unequal variances was used to compute *P* values. Differentially expressed genes were considered to be significant when *P* ≤ 0.01 and |FC| ≥ 2. Expression values of differentially expressed genes were then centered on the median (z-scores), and heat maps were produced using MeV 4.8.1 software^96^. Unsupervised hierarchical clustering analysis (HCA) of the differentially expressed genes was also performed in MeV 4.8.1. Clustering was done with complete linkage and Euclidean distance. Venn diagrams were performed in Venny 2.1 (http://bioinfogp.cnb.csic.es/tools/venny/index.html). To test the significance of overlap in Venn diagrams, the hypergeometric tests were performed in R, using the *dhyper* function from the stats package. Population size was considered to be 60,483, the total number of genes for which RNA-seq data were available in TCGA.

### Functional enrichment analysis

Gene ontology (GO) functional categories were analyzed using DAVID^98^ or WebGestalt^99^ software packages. Pathways enrichment was investigated using the WebGestalt software packages. KEGG and Pathway Commons databases were screened. Enrichment of DNA binding sites was also investigated though WebGestalt using the Transfac database. Bonferroni-adjusted *P* values of the hypergeometric test were used to determine enrichment significance. Geneset enrichment analysis was performed using GSEA v2.0.14 software with 1000 phenotype permutations^100^. Gene sets were downloaded from MSigDB^101^. Enrichment maps were generated with the Enrichment Map Plugin 1.3^102^ developed for Cytoscape 2.8^103^ using the default parameters.

### Availability of data and materials

All data used in this work are available through the following databases: ONCOMINE (https://www.oncomine.org/*)*, ATCG (https://cancergenome.nih.gov/), cBioportal (http://www.cbioportal.org/) and PrognoScan databases (http://www.abren.net/PrognoScan/).

## Electronic supplementary material

Supplementary Figures and Tables

Supplementary Table S3

Supplementary Table S4

Supplementary Table S5

Supplementary Table S6

Supplementary Table S7

Supplementary Table S8

Supplementary Table S9
