# Supplementary material for: High expression of SMARCA4 or SMARCA2 is frequently associated with an opposite prognosis in cancer
Source: Sci Rep. 2018 Feb 1;8:2043. doi: 10.1038/s41598-018-20217-3 (PMC5794756; doi:10.1038/s41598-018-20217-3)
Supplement: Supplementary file 1 — Supplementary Figures and Tables [file 41598_2018_20217_MOESM1_ESM.pdf]

High expression of *SMARCA4* or *SMARCA2* is frequently associated with an opposite prognosis in cancer

Jose A. Guerrero-Martínez and Jose C. Reyes

Supplementary Figures and Tables.

Supplementary Table S1
Supplementary Table S2
Supplementary Figure S1
Supplementary Figure S2
Supplementary Figure S3
Supplementary Figure S4
Supplementary Figure S5
Supplementary Figure S6

Table S1. Datasets with significant changes of *SMARCA4* expression in tumors versus normal tissue. Data obtained from ONCOMINE.

Type of Tumor	Fold change [a]	*P* value	N normal	N tumor	Data Link
Pleural Malignant Mesothelioma	4.091	5.73E-6	4	40	GSE2549
Cutaneous Melanoma	4.051	2.93E-7	7	45	GSE3189
Smoldering Myeloma	4.035	1.30E-6	22	12	GSE5900
Monoclonal Gammopathy of Undetermined Significance	2.269	5.86E-10	22	44	GSE5900
B-Cell Acute Lymphoblastic Leukemia	3.183	2.29E-66	74	174	GSE13159
Pro-B Acute Lymphoblastic Leukemia	2.465	2.60E-33	74	70	GSE13159
T-Cell Acute Lymphoblastic Leukemia	2.460	1.00E-53	74	174	GSE13159
B-Cell Childhood Acute Lymphoblastic Leukemia	2.385	1.02E-63	74	359	GSE13159
Superficial Bladder Cancer	3.172	1.56E-16	48	28	PMID:16432078
Infiltrating Bladder Urothelial Carcinoma	2.320	8.35E-13	48	81	PMID:16432078
Hepatocellular Carcinoma	3.038	2.43E-9	10	35	GSE6764
Primary Effusion Lymphoma	2.815	1.07E-6	5	9	GSE2350
Colorectal Carcinoma	2.688	3.66E-13	12	70	GSE9348
Hepatocellular Carcinoma	2.663	4.09E-9	21	22	GSE14520
Anaplastic Large Cell Lymphoma	2.621	5.23E-12	10	6	GSE6338
Myxofibrosarcoma	2.515	6.70E-10	9	31	GSE21122
Pleomorphic Liposarcoma	2.385	1.16E-7	9	23	GSE21122
Leiomyosarcoma	2.045	1.86E-7	9	26	GSE21122
Myxoid/Round Cell Liposarcoma	2.040	1.37E-7	9	20	GSE21122
Infiltrating Bladder Urothelial Carcinoma	2.375	1.71E-7	9	13	GSE3167
Rectal Adenoma	2.156	1.03E-5	7	7	GSE8671
Hepatocellular Carcinoma	2.149	6.01E-56	220	225	GSE14520
Colon Mucinous Adenocarcinoma	2.116	3.88E-6	5	13	GSE5206
Astrocytoma	2.088	6.22E-5	7	5	PMID:12894235
Glioblastoma	2.011	3.79E-5	7	27	PMID:12894235
Plasma Cell Leukemia	2.074	8.72E-5	5	9	GSE13591
Invasive Breast Carcinoma Stroma	-20.023	9.71E-27	6	53	GSE9014
Chronic Lymphocytic Leukemia	-2.717	3.12E-8	5	34	GSE2350
Teratoma, NOS	-2.715	2.03E-10	6	14	GSE3218
Glioblastoma	-2.373	1.59E-7	3	22	GSE4536
Smoldering Myeloma	-2.158	3.29E-6	22	12	GSE5900
Esophageal Adenocarcinoma	-2.012	7.57E-12	28	75	GSE13898

[a] Linear Fold-Change tumor versus normal tissue. Positive values indicate increase of expression, negative values indicate decrease of expression.

Table S2. Datasets with significant changes of *SMARCA2* expression in tumors versus normal tissue. Data obtained from ONCOMINE.

**Type of Tumor**	**Fold change**[a]	***P* value**	**N normal**	**N tumor**	**Data Link**
Benign Melanocytic Skin Nevus	4.137	9.56E-8	7	18	GSE3189
Smoldering Myeloma	3.386	4.39E-9	22	12	GSE5900
Chronic Lymphocytic Leukemia	2.336	3.68E-5	6	3	PMID:12075054
Invasive Breast Carcinoma	2.256	5.83E-12	6	53	GSE9014
Yolk Sac Tumor, NOS	-12.346	2.48E-11	6	9	GSE3218
Mixed Germ Cell Tumor, NOS	-4.278	2.41E-11	6	41	GSE3218
Embryonal Carcinoma, NOS	-4.189	9.73E-9	6	15	GSE3218
Ovarian Serous Adenocarcinoma	-4.573	3.50E-11	10	43	GSE12470
Classic Medulloblastoma	-4.370	3.18E-6	4	46	PMID:11807556
Ovarian Carcinoma	-4.364	8.08E-8	10	185	GSE26712
Hepatocellular Carcinoma	-2.715	3.01E-8	21	22	GSE14520
Lung Adenocarcinoma	-2.507	3.12E-15	10	86	PMID:12118244
Hepatocellular Carcinoma	-2.216	1.41E-6	10	35	GSE6764
Ductal Breast Carcinoma	-2.207	5.98E-11	7	40	GSE3744
Glioblastoma	-2.107	1.62E-12	4	27	GSE2223
Ovarian Serous Adenocarcinoma	-2.063	6.67E-6	4	41	GSE6008

[a] Linear Fold-Change tumor versus normal tissue. Positive values indicate increase of expression, negative values indicate decrease of expression.

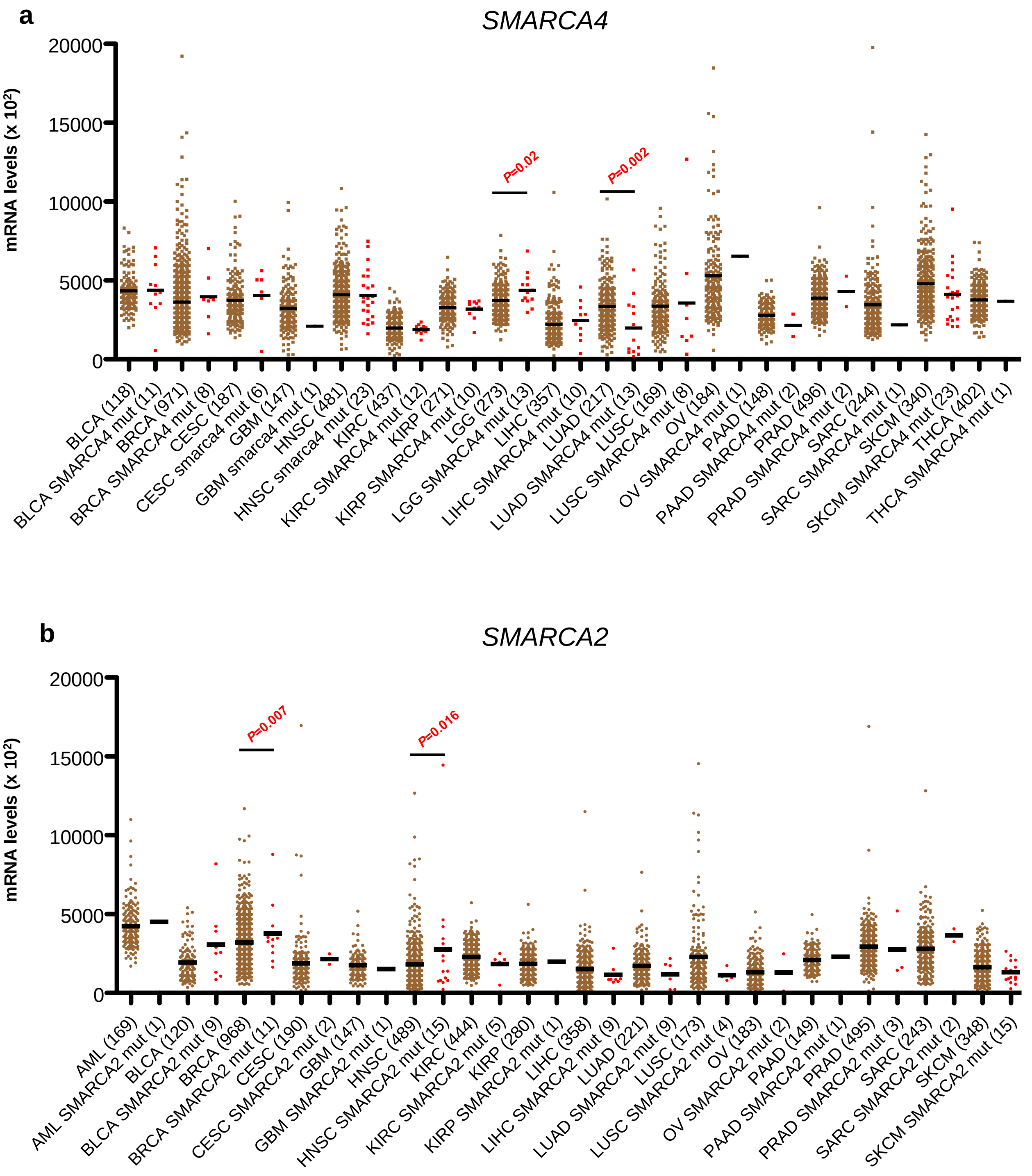

Figure S1. Levels of *SMARCA4* and *SMARCA2* expression in multiple types of tumors. a, b Effects of gene mutation on the levels of *SMARCA4* (**a**) or *SMARCA2* (**b**) mRNA (RNA-seq data) in samples of difference tumor types (data obtained from TCGA). Data from tumors with mutated *SMARCA4* or *SMARCA2* are depicted in red. Data from cohorts in which none of the tumors presented mutations in the *SMARCA4* or *SMARCA2* gene were not included in the graph. Number of tumors of each set is provided (note that the number of tumors does not always match the data given in Additional file 2: Table S3 due to the absence of sequencing data). Student´s t-test *P* value ($P \leq 0.05$) are provided.

Figure S1

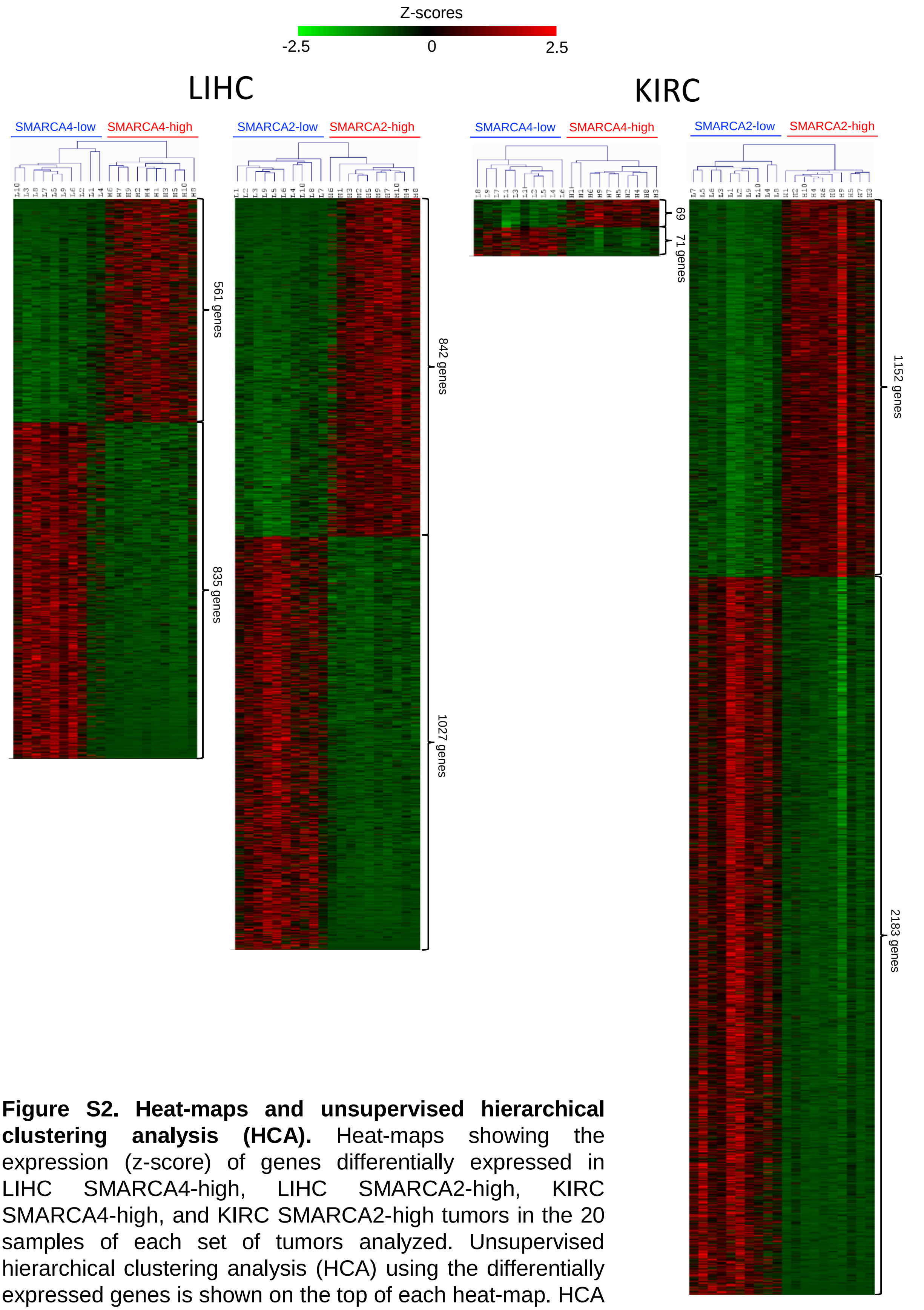

Figure S2. Heat-maps and unsupervised hierarchical clustering analysis (HCA). Heat-maps showing the expression (z-score) of genes differentially expressed in LIHC SMARCA4-high, LIHC SMARCA2-high, KIRC SMARCA4-high, and KIRC SMARCA2-high tumors in the 20 samples of each set of tumors analyzed. Unsupervised hierarchical clustering analysis (HCA) using the differentially expressed genes is shown on the top of each heat-map. HCA separated SMARCA4-high from SMARCA4-low tumors and SMARCA2-high from SMARCA2-low tumors in all the cases.

Figure S2

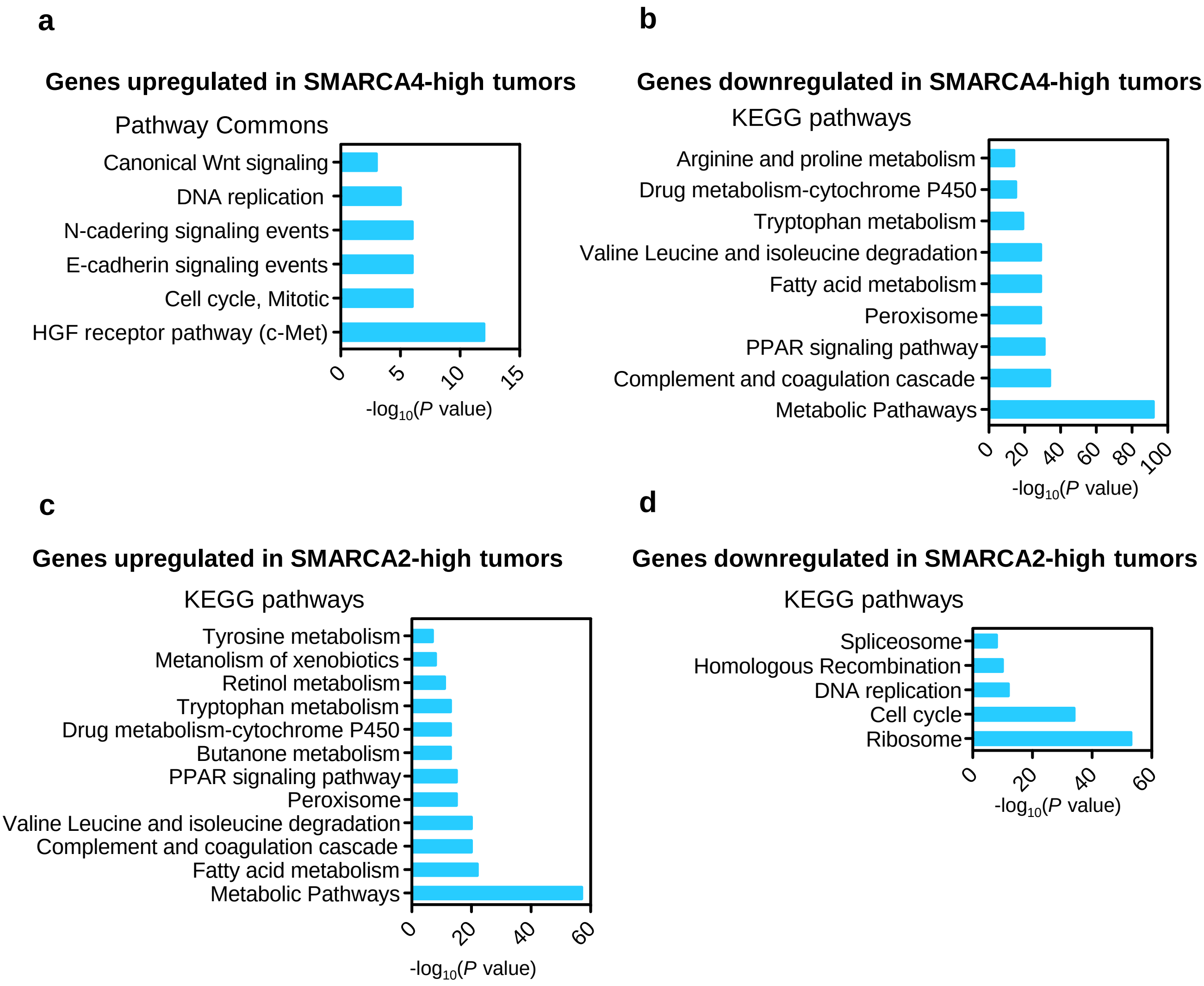

Figure S3. Pathway enrichment data. Genes differentially expressed in LIHC SMARCA4-high and SMARCA2-high tumors were analyzed for pathway enrichment using WebGestalt software packages. KEGG database pathways are provided, except for SMARCA4-high, for which the KEGG pathway enrichments were not significant and the Pathway Commons database was screened instead. Bonferroni-adjusted *P* values of the hypergeometric test were used to determine enrichment significance.

Figure S3

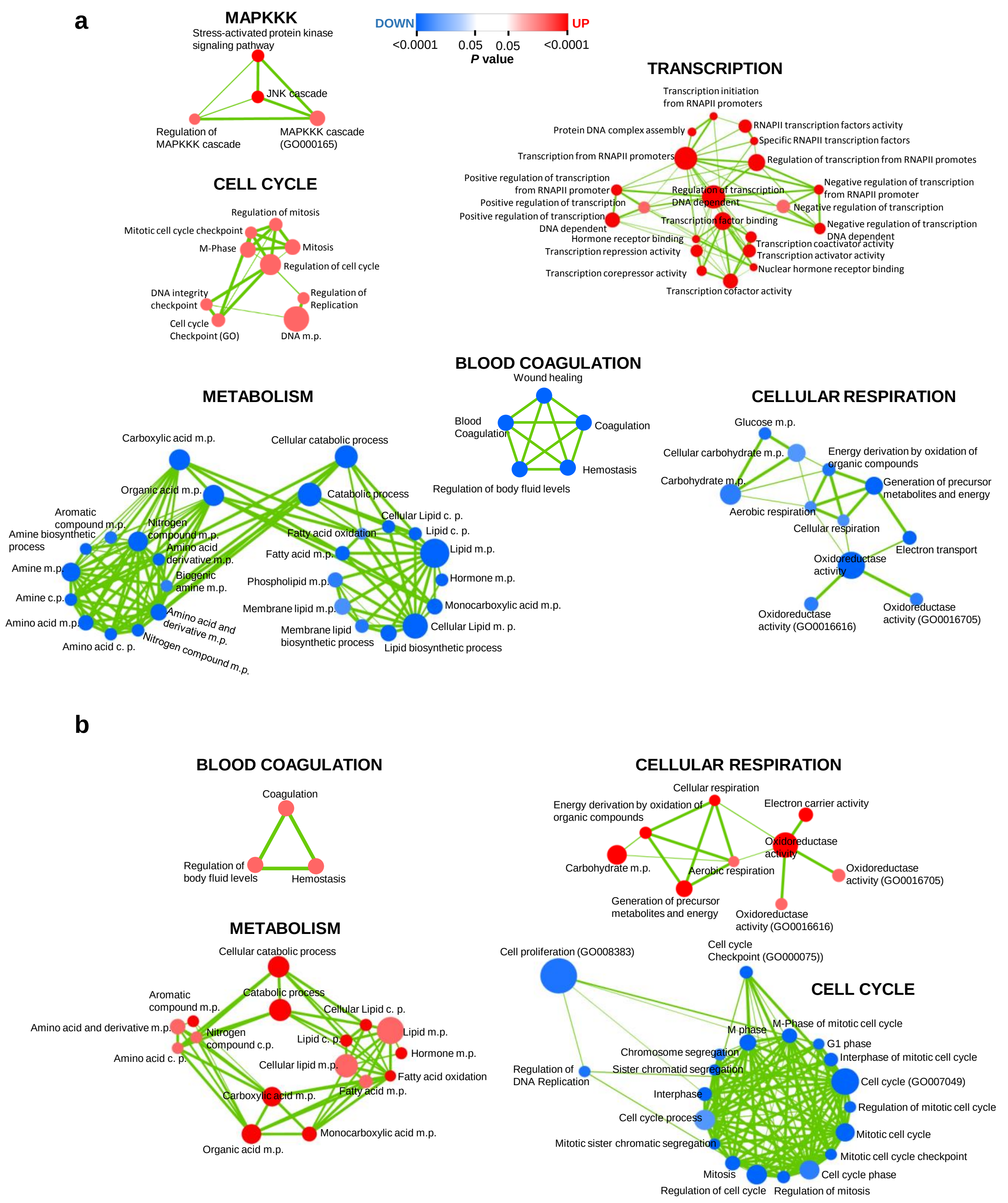

Figure S4. GSEA enrichment maps of genes differentially expressed in LIHC SMARCA4-high (a) and SMARCA2-high (b) tumors. a, b Geneset enrichment analysis was performed using GSEA v2.0.14 software with 1000 phenotype permutations. Nodes corresponded to enriched MSigDB gene sets. Node size is proportional to the number of matched genes in the geneset. Upregulated functions (nodes) are depicted in red, and downregulated functions, in blue. Node color intensity is proportional to the GSEA *P* value, and edge thickness, to the number of overlapping genes between categories. m.p. metabolic process; c.p. catabolic process

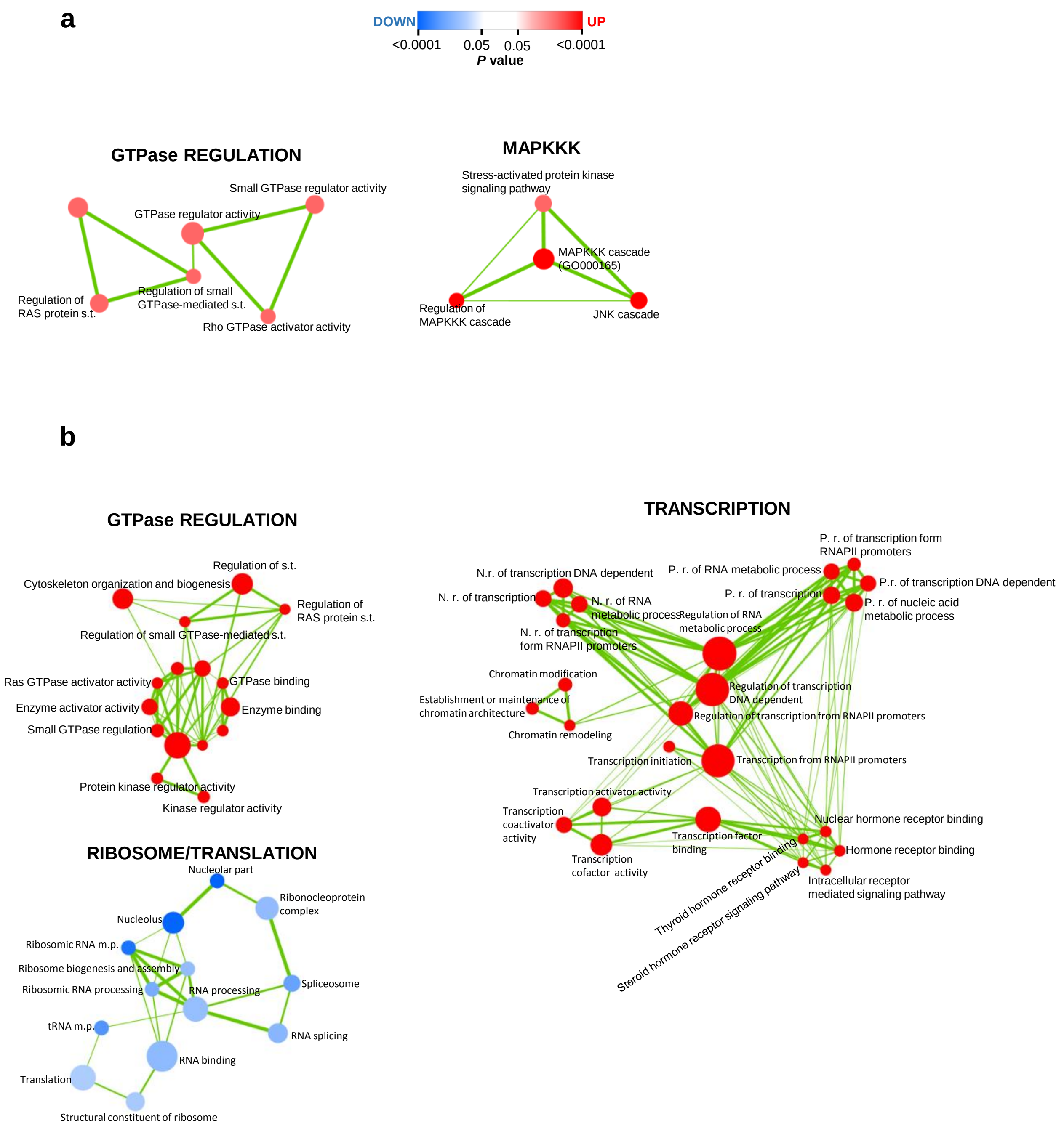

Figure S5. GSEA enrichment maps of genes differentially expressed in KIRC SMARCA4-high (a) and SMARCA2-high (b) tumors. See Additional file 3: Figure S4 legend for details. m.p. metabolic process; s.t. signal transduction; N.r. negative regulation; P.r. Positive regulation.

Figure S5

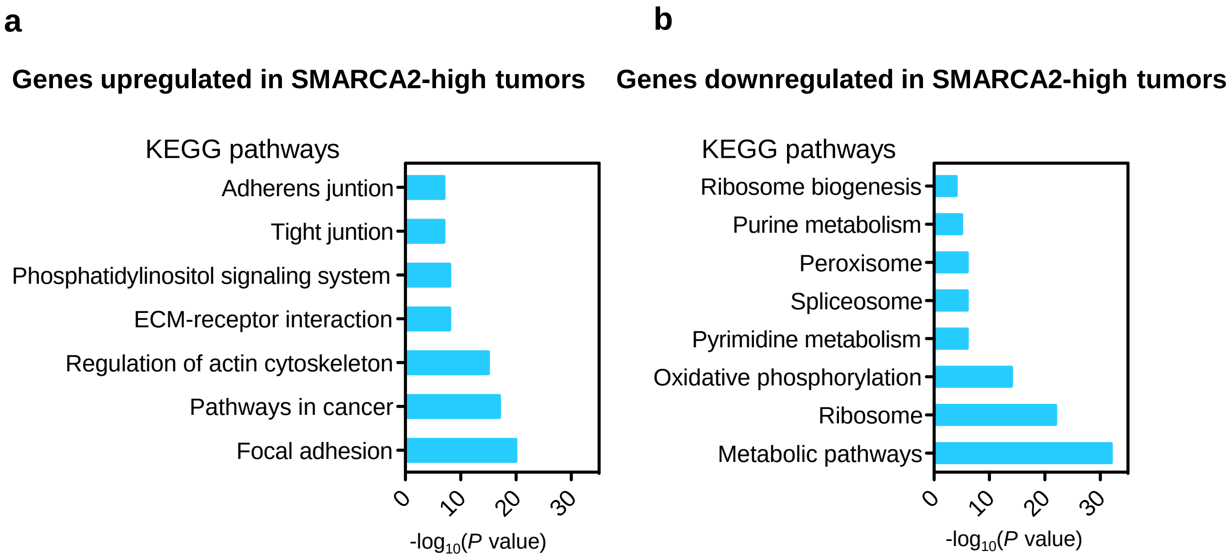

Figure S6. Pathway enrichment data. Genes differentially expressed in KIRC SMARCA4-high and SMARCA2-high tumors were analyzed for pathway enrichment using WebGestalt software packages. Bonferroni-adjusted *P* values of the hypergeometric test were used to determine enrichment significance.

Figure S6
